# Unraveling Neurological Shades: Vitamin D Toxication and Central Pontine Myelinolysis Exposed

**DOI:** 10.7759/cureus.53806

**Published:** 2024-02-07

**Authors:** Abhinav Ahuja, Sachin Agrawal, Varun Daiya, Nitish Batra, Aaditi Agarwal

**Affiliations:** 1 Department of Medicine, Jawaharlal Nehru Medical College, Datta Meghe Institute of Higher Education and Research, Wardha, IND

**Keywords:** central pontine myelinolysis, hypervitaminosis, neurological symptoms, dehydration, hyponatremia

## Abstract

This case report is about a middle-aged female who presented with complaints of pain in the abdomen with intractable vomiting for three months, pain and weakness in bilateral lower limbs for two months, and irritability for three days. She was previously treated for lumbar disc bulge and severe narrowing of the spinal cord whose treatment also included vitamin D supplements. After taking high doses of a vitamin D supplement daily for approximately four months, it resulted in vitamin D toxicity. The sodium level of the patient was in the normal range throughout the treatment. Her magnetic resonance imaging brain revealed features of central pontine myelinolysis. The development of central pontine myelinolysis due to vitamin D toxicity, with normal sodium levels, makes this a rare case for discussion.

## Introduction

Damage to the brain's myelin sheath, which shields neurons, is the hallmark of the clinical disorder known as central pontine myelinolysis, which most frequently affects the pontine white matter tracts [1]. This disorder mainly occurs in patients with hyponatremia, hypokalemia, low Glasgow Coma Scale (GSC), chronic malnutrition, chronic liver disease, alcoholic patients, severe cases of burns, or hyperemesis gravidarum. The primary etiology of central pontine myelinolysis is hyponatremia, which is corrected quickly. The patient commonly presents with altered sensorium, hyperemesis, abdominal pain, quadriplegia, or other neurological symptoms. Patients with central pontine myelinolysis often have a biphasic course, the first phase reflecting the underlying predisposing illness and the second phase reflecting pontine dysfunction, impaired vigilance, and movement disorders, among other neurological abnormalities [2].

Vitamin D is an important mineral that is a major source of growth and strength of the bones. This is an over-the-counter drug, taken as a supplement to overcome its deficiency. Vitamin D toxicity occurs due to excessive consumption or inaccurate prescriptions for the supplements. This results in an imbalance in bone metabolism which leads to the development of hypercalcemia [3]. The overall incidence of neuronal damage is very low and reversible in the early stages of the disorder, but delayed treatment can cause permanent damage [4].

## Case presentation

A 52-year-old female patient presented to the emergency department with complaints of altered sensorium with agitation for three days, weakness and pain in bilateral lower limbs for two months, and abdominal discomfort with persistent vomiting for three months. The patient has a history of taking treatment for lumbar disc bulge at the level of L2 to L5 and S1 and a narrowing of the right neural foramina along with severe narrowing of the spinal canal from L3 to L5 and straightening of the cervical spine. The treatment mainly included vitamin D injectable supplements.

The patient had consumed excessive amounts of the supplement, which resulted in excruciating chest and abdominal discomfort and frequent vomiting episodes which were not relieved on medication. An endoscopy was done which was suggestive of antral gastritis with rapid urease test positive. She was a known case of hypertension, diabetes mellitus, and hyperthyroidism for two years and is currently on Tablet Telmisartan 40 mg OD, Tablet Metformin 500 mg BD, and Tab Neomercazole 10 mg TDS, respectively.

There was no history of fever, cough, cold, loss of consciousness, trauma, bronchial asthma, or tuberculosis. The physical examination revealed that the patient appeared to be of average build. There was some evident mild abdominal tenderness. The patient had a GCS score of 9 and seemed sluggish and unresponsive to orders. The blood pressure at the time was 150/90 mm Hg, and the pulse rate was 110 beats per minute and was regular. As indicated in Table 1, laboratory tests were performed for the total blood count, HBA1c, anti-thyroglobulin antibody, antinuclear antibody, and kidney function test. The hypercalcemia raised the possibility of multiple myeloma. To confirm the diagnosis, serum protein electrophoresis testing was carried out. The results showed an absence of an M band in the gamma region of serum protein electrophoresis, which is a common marker for multiple myeloma. Additionally, the chromatography was found to be normal, indicating no abnormalities in the protein distribution.

**Table 1:** Investigations done and laboratory reference values.

S. NO.	INVESTIGATIONS	OBSERVED VALUE	EXPECTED VALUE
1	Haemoglobin (gm%)	12.2	12-16
2	White blood cells (cu..mm)	10200	4000-11000
3	Procalcitonin (ng/ml)	2.2	< 0.05
4	HBA1c	5.7	≤ 5.6
5	Serum Urea (mg/dL)	40	6-24
6	Serum Creatinine (mg/dL)	1.68	0.7-1.2
7	Serum Sodium (mEq/L)	138	131-145
8	Serum Potassium (mmol/L)	4.1	3.6-5.2
9	Anti-thyroglobulin antibody (IU/ml)	17.3	< 60
10	Anti-Nuclear Antibody	Negative	Negative
11	Serum Protein Electrophoresis	Normal	Normal
12	Intact PTH (pg/mL)	8.9	15-65
13	serum calcium	14	8-10
14	Serum ionic calcium (mg/dL)	5.2	4.65-5.25
15	Thyroid-stimulating hormone (mlU/L)	2.6	0.5-5.0
16	Free T3 (pg/mL)	3.12	2.3-4.1
17	Free T4 (pg/mL)	11.16	9.0-17.0
18	Serum Vitamin D (mmol/L)	140	<50

**Figure 1:**
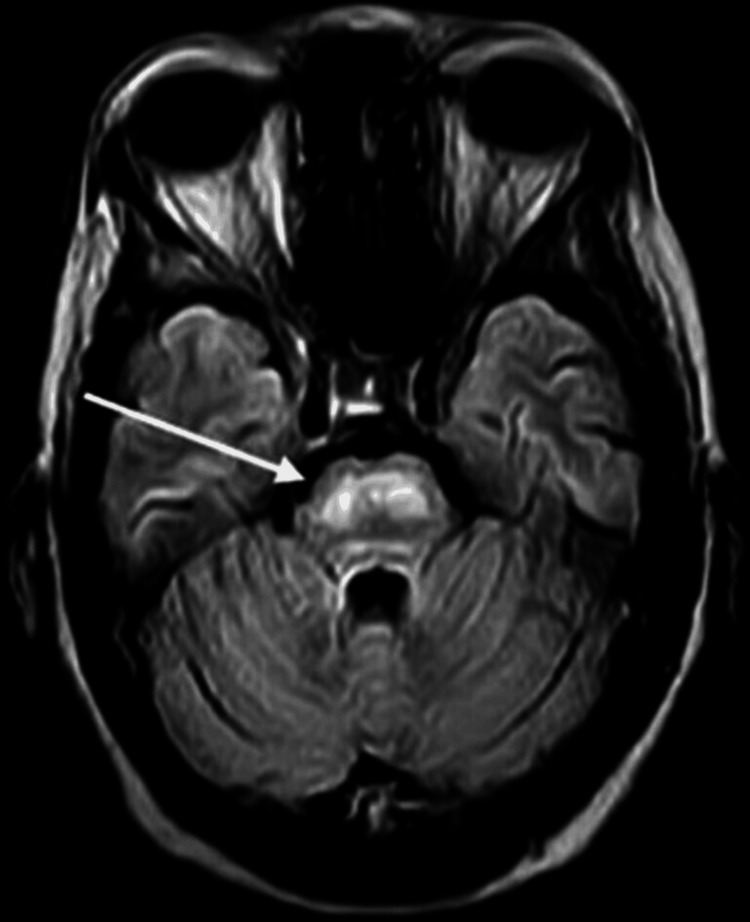
Persistence of abnormal T2/flair hyperintensity centrally in pons with peripheral sparing on magnetic resonance imaging.

On magnetic resonance imaging (MRI) brain, there is persistence of abnormal T2/flair hyperintensity centrally in pons with peripheral sparing which is consistent with demyelination as shown in Figure 1.

The patient received a 100-unit injection of calcitonin to lower blood calcium levels, aid in lowering vitamin D levels, and relieve neurological symptoms. With calcitonin injections, the patient's symptoms significantly improved. The patient was given a two-week treatment plan. Continuous monitoring of vital signs was conducted alongside repeated blood investigations, including tests for thyroid function and blood glucose. The results of the tests were all within the normal range. Following a successful treatment, the patient made a complete recovery and was discharged. She was doing well on follow-up after one month.

## Discussion

Vitamin D toxicity can occur in a patient who has a history of consumption of vitamin D supplements more than the recommended daily allowance dose. The lever of 25-hydroxycholecalciferol becomes >150 ng/mL. In newborns, 1,000 mcg (40,000 units) of vitamin D daily leads to toxicity in one to four months. Adults can develop toxicity after consuming 1,250 mcg (50,000 units) each day for several months. Patients who have hypercalcemia also have vitamin D toxicity. Overcorrection of hypoparathyroidism can cause iatrogenic vitamin D toxicity. The symptoms include nausea, vomiting, anorexia, anxiety, weakness, polyuria, polydipsia, and eventually renal failure. The main aim of treatment is to reduce serum calcium levels in the blood which is done by IV saline infusion which corrects dehydration and increases renal clearance of calcium, also bisphosphonates are given which inhibit resorption of the bones [5]. Co-administration of calcitonin and bisphosphonates is done to increase the effectiveness of the drugs [6].

In this case report, the patient had a history of treatment for spondylosis six months ago with a lumbar disc bulge and severe narrowing of the spinal cord. She was prescribed supplementary injections for vitamin D to aid the healing process which led to the intoxication of vitamin D in the blood leading to acute gastrointestinal symptoms and was presented in a state of altered sensorium [7].

Central pontine myelinolysis is a rare disorder where damage to the neurons of the brain occurs due to myelinolysis. It can occur in any age group. The primary cause of this is the quick repair of sodium electrolytes in hyponatremia patients. Myelinolysis is a serious condition that can occur when there is a rapid influx of sodium ions. This influx prevents neurons from absorbing enough sodium ions, ultimately leading to damage [8]. The peripheral sodium concentration becomes relatively higher than the brain which leads to the pulling of water molecules too. This leads to dehydration which further adds up to myelinolysis. The cause of this disorder in this patient is due to vitamin D toxicity which was given to the patient as an injectable supplement to aid adequate healing for the surgery of lumbar disc prolapse.

A retrospective study, conducted in 2015, documented the incidence of central pontine myelinolysis to be 2.5% among patients admitted to intensive care unit [1]. Central pontine myelinolysis has been reported to have a poor prognosis, with death rates as high as 40%-50%. A retrospective study focusing on individuals suffering from this illness conducted in India by Rao et al. found that 18% of patients were in a vegetative state and that 12% of patients died [5].

The aim of the treatment for this disorder is primarily to prevent further damage to the myelin sheath of the neurons of the brain. The most common cases of central pontine myelinolysis require mainly sodium and water correction in the system whereas in this case, the patient's vitamin D toxicity was corrected using calcitonin injection while stopping calcium supplementation. For correction, initially, isotonic saline is infused into the body for proper rehydration and to increase the renal clearance of calcium. About 800 mg of calcium are eliminated in the feces and 200 mg in urination for every gram of calcium consumed through meals [5]. Along with rehydration, calcitonin is injected in a concentration of 4 U/kg intramuscularly every 12 hours for 48 hours. The co-administration of bisphosphonates and calcitonin has proved to increase the efficacy of calcitonin [9]. Calcitonin is an inhibitor of osteoclastic activity of the bone hence inhibits bone resorption. The patient responded positively to the treatment with calcitonin and subsequently the symptoms were treated.

The differentials for central pontine myelinolysis include hyperthyroid encephalopathy, multiple myeloma, acute infectious encephalopathy, and CNS vasculitis. Central pontine myelinolysis may cause severe neurological damage which may further lead the patient into coma or death. Secondary complications may develop with increasing severity of the illness which includes venous thromboembolism, and pulmonary infections for which the patient may need ventilatory support [10].

## Conclusions

Central pontine myelinolysis is a rare neurological disorder that destroys the myelin sheath in the brainstem. Severe electrolyte imbalances often accompany this condition, which must be monitored closely to prevent complications. Early diagnosis and treatment are crucial in preventing significant mortality and morbidity. Treatment may comprise electrolyte replacement therapy and medications to manage symptoms. Closely monitoring patients to ensure a positive response to treatment and prompt addressing of complications is essential.
